# Land‐use changes interact with geology to facilitate dispersal of the rock hyrax (*Procavia capensis*) and leishmaniasis across Israel and the West Bank

**DOI:** 10.1002/ece3.9915

**Published:** 2023-03-21

**Authors:** Noam Ben‐Moshe, Marcelo Rosensaft, Takuya Iwamura

**Affiliations:** ^1^ Tel Aviv University, Zoology Tel Aviv Israel; ^2^ Geological Survey of Israel Jerusalem Israel; ^3^ Department F.‐A. Forel for Aquatic and Environmental Sciences and Institute for Environmental Sciences University of Geneva Geneva Switzerland

**Keywords:** geology, land‐use change, rock hyrax

## Abstract

Geology plays a fundamental role in establishing species' habitats, determining both physical (e.g., landscape morphology, soil texture) and chemical (e.g., mineral composition, water availability) properties. In the current Anthropocene epoch, human activity is transforming Earth's geology and ecosystems. Yet to date, there have been almost no studies incorporating geology when examining the effect of such land‐use changes on species distribution. This study seeks to uncover how specific land‐use changes interact with geology, in order to explain the recent and rapid expansion of the rock hyrax (*Procavia capensis*) across the mountains of central Israel and the West Bank. Hyraxes are dependent on rock mounds for their habitat, and their expansion seems to be correlated with increasing infrastructure construction. However, their expansion patterns differ among locations, even when the human land‐use is similar. To explain the patterns of hyrax distribution observed over the past 46 years, we converted geological data into ecological data, which present the probability of the local bedrock breaking into boulders, whether due to either natural or anthropogenic weathering processes. We applied species distribution models (SDMs) and found that the expansion of rock hyrax populations was facilitated by means of the interaction of specific geological units with land‐use practices (e.g., roads and construction), which resulted in the accumulation of large boulders, creating novel habitats and stepping stones in previously unsuitable areas for hyraxes. Since rock hyraxes are major hosts of the leishmaniasis pathogen, the findings from this study offer important insights into the progression and potential outbreaks of the disease in human populations. Understanding the role that geology plays in shaping a species' niche is expected to prove useful in studying the distribution of other wildlife species and is fundamental in studies seeking to predict the potential ecological impacts on local biodiversity associated with land‐use change.

## Graphical abstract

Geology can play a significant role in dispersal ability of animals, especially when it interacts with land use, a subject that has received less study. We examined the causes of recent expansion of the rock hyrax, an important host of leishmaniasis, across Israel and the Palestinian Territories based on the distribution of geological units and human development. We demonstrate that rock hyrax expansion is facilitated where roads and construction intersect with specific rock formations, resulting in rock mounds that serve as new habitats for hyraxes.

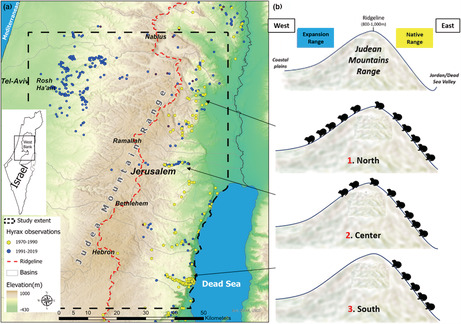

## 1 INTRODUCTION

Geodiversity is strongly tied with biodiversity (Kruckeberg, 2002). The mineral composition of the geological substrate determines the nutrients available to plants (Morford et al., 2011) and the toxin concentrations (Brady et al., 2005). The physical properties of the bedrock govern its water storage capacity, cohesion and stability (Egerton‐Warburton et al., 2003), while its resistance to erosion contributes to the morphology and topography of the landscape (Hahm et al., 2014; Kruckeberg, 2002; Ott, 2020), which can further affect fine‐scale climatic conditions (Caputa, 2016; Yarwood et al., 2020). Geology, therefore together with climate, provides some of the essential elements of the ecological niches, thereby determining the species that are able to inhabit a particular area (Antonelli et al., 2018; Hahm et al., 2014; Moriarty & Honnery, 2004; Ott, 2020).

The *Anthropocene* has been proposed as the latest geological epoch (Finney & Edwards, 2016; Zalasiewicz et al., 2019), in which anthropogenic forces are transforming the planet's surface and affecting plants and animals by altering their habitat conditions (García‐Quintana et al., 2004; Jetz et al., 2007; Koellner & Scholz, 2008; Laurance et al., 2018; Lawler et al., 2014). Although anthropogenic land‐use changes often negatively impact wildlife through habitat loss and fragmentation (Laurance et al., 1997; Sala et al., 2000), they may also facilitate the dispersal of some species through the creation of novel habitats analogous in form or function to natural ones (Balbontín et al., 2008; Lundholm & Richardson, 2010; Mendelssohn & Yom‐Tov, 1999). Such habitats allow animals to move across otherwise unhospitable terrain (Gherghel et al., 2009) or beyond the species' preferred climatic range (Cannizzo Id & Griffen, 2019). With the ongoing and intensifying global land‐use processes (Lawler et al., 2014), understanding and predicting the impacts of these changes on wildlife distributions are urgent scientific priorities for conservation (Rosenzweig, 2003; Sol et al., 2013) and for the wellbeing of both wildlife (Bennett, 2017; Forman & Alexander, 1998; Lepczyk et al., 2004; Wise, 2007) and humans (Daszak et al., 2000; Messmer, 2000; Patz et al., 2004; Soulsbury & White, 2015). Human land‐use and geology are interwoven, both spatially and causally. Geology, on one hand, determines the locations, boundaries and numerous types of land‐uses, such as agriculture, mining and quarrying, which are dependent on the suitable properties of the processed soils (White, 2003) or types of rock extracted (Gunn & Bailey, 1993). In modern urban land‐use planning, geology constitutes the central database for undertaking suitability analyses for human settlement areas, particularly in rapidly developing countries such as China (Dai et al., 2001). On the other hand, land‐uses also affect the geology itself: for example, agricultural land‐use can alter the soil's chemical and physical properties such as pH (Balstrøm et al., 2013), mineral composition and bulk densities (Pouyat et al., 2007), while excavation activities (mining and construction) can expose and move buried rocks and soils, converting them into a growing medium for plants (Hooke, 2000; Wali, 1999).

Surprisingly, the combined impacts of geology and land‐use change have been mostly overlooked in ecological studies (Wiggering, 2014), despite such interactions having a potentially substantial effect on biodiversity. For example, agriculture and mining affect aquatic ecosystems through soil erosion, leading to changes in species abundance and composition (Alin et al., 2002; Shearer & Young, 2011). Stone quarries have a unique morphology and mineral content that generate new habitats, favored by some plant and wildlife species (Germano et al., 2016; Telea et al., 2019).

**FIGURE 1:**
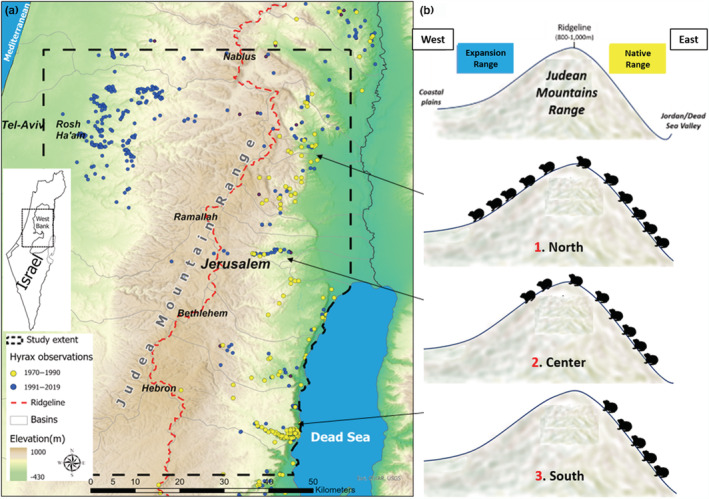
Study areas and movement of Rock hyrax. (a) Map of the study area indicating hyrax observations across the Judean Mountain range of central Israel and the Palestinian Territories (Observation records were provided by Israel's National Parks Authority). Until 1990, hyraxes were observed (yellow dots) only to the east of the ridgeline (dashed red line) but expanded to the western slopes ever since (blue dots). The expansion pattern is different between three regions: hyraxes expanded approximately 40 kilometers west of the ridgeline in the north, and 4 kilometers in the central region, but did not expand in the south. (b) Cross‐sectional schematic of the hyrax dispersions across the Judean Mountains Range. Northern and central populations cross the ridge while southern population did not.

Rock hyraxes (*Procavia capensis*) are medium‐sized mammals that inhabit crevices in rock mounds or outcrops, where they find refuge from predators and extreme weather conditions (Hoeck & Bloomer, 2003). Native to Israel and the Palestinian Territories, their distribution has been expanding in the last few decades, correlated to land‐use changes associated with rock excavation and the creation of artificial rock piles (Ben‐Moshe & Iwamura, 2020; Mendelssohn & Yom‐Tov, 1999; Moran et al., 1987; Salah et al., 2020; Waitz et al., 2019). Their expansion near human settlements is considered a risk to human health as the hyrax is a reservoir host of *Leishmania tropica*, a pathogenic protozoon causing leishmaniosis which can be transmitted to humans via sandflies (Jaffe et al., 2004; Talmi‐Frank et al., 2010). This increased risk to human health necessitates an understanding of the hyrax dispersion dynamics in order to designate effective control measures. The Judean Mountains of the West Bank have become a focal expansion area for hyraxes, which have dispersed from their historical range on the sparsely human‐populated eastern slopes to the densely populated ridge and western slopes (Figure 1). However, despite the seemingly similar climatic and terrain conditions along their expansion routes across the ridge, observation records (see Section 2.3.1) indicate that the hyrax populations have expanded at varying rates at different regions of the mountains.

**FIGURE 2:**
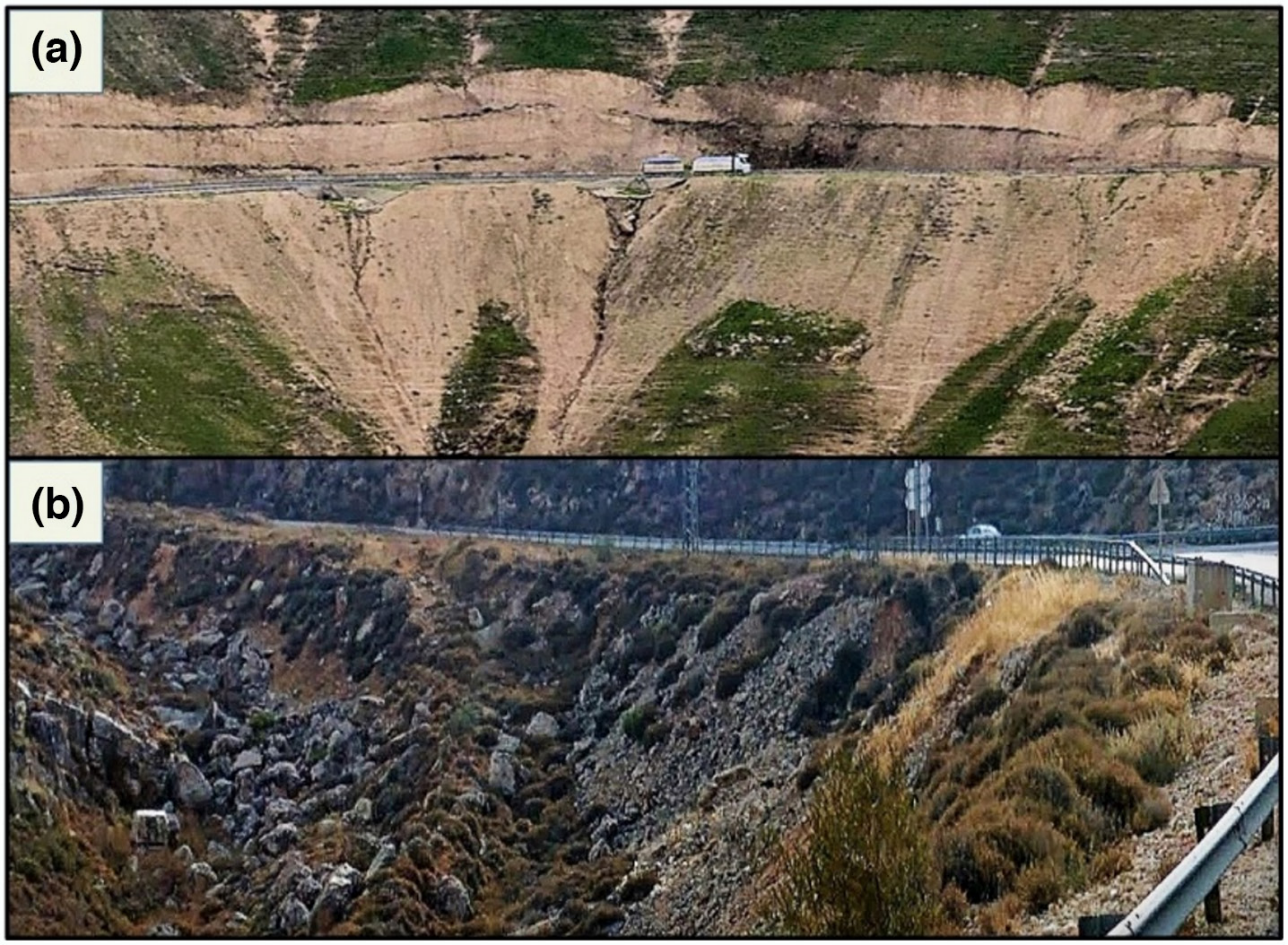
Debris formed on roadsides carved into different geological formations has different potentials for sheltering the rock hyrax. (a) Debris made of small particles alongside highway 1 carved into soft rock formation. (b) Large boulders formed along highway 5 carved into hard rock formation.

We suggest that these differences in expansion patterns represent differences in shelter availability and dispersal possibilities, governed mainly by geology–land‐use interactions (see example in Figure 2).

We hypothesized that the hyrax expansion patterns are modified by a combination of two key factors: (1) geological diversity, in which only specific rock formations are able to break down into large boulders suitable for hyraxes and (2) an external force able to break up the rocky foundation. Such force can be natural—erosion in steep areas or geological faults, or due to anthropogenic land‐use change—such as infrastructure construction, road paving or quarrying. We predicted that novel habitats created by the interaction of geology with land‐use change would create stepping‐stone refugia that enable the hyrax to cross the mountains and disperse on the western slopes. Based on our assumptions, we developed a method to exploit geological data to define areas that have the potential to form hyrax habitats. We combined this with environmental and land‐use change data, to determine how these affect the expansion patterns revealed by the hyrax observation records. The results provide further insight into a relatively little studied aspect of the interaction between geology and land‐use change.

## 2 METHODS

### 2.1 The study area

The study area encompasses 6000 km^2^ in the West Bank area of central Israel and the Palestinian Territories. This region has become a major hotspot of leishmaniosis in recent years (Jaffe et al., 2004). The area is located along a longitudinal ridge ([−]400–1000 m above sea level, Figure 1a). Climatically, it is an ecotone between a Mediterranean climate on the western side of the ridge and arid eastern slopes.

It is a densely populated region with approximately 3 million inhabitants, with most of the population living on the western slopes and the upper parts of the ridge. Land‐use change has been dramatic in the last 40 years, reflecting the high‐population growth rate of the Palestinians and the establishment of numerous Israeli settlements (Tal, 2016).

In terms of geology and on a regional scale, the Judean Mountains are not geologically diverse, comprising mainly a carbonate sequence, composed mostly of limestone and dolomite, with minor components of chalk, marl and chert (Israel Geological Survey). However, a fine resolution mapping of the area (1:50,000; Israel Geological Survey), presenting the geological formations, revealed that the area has a diverse lithology and exposure pattern. In particular, natural and anthropogenic weathering processes have formed different types of slope debris that vary from fine particles to large boulders. This is an important observation because, for the rock hyraxes, only large boulders with wide crevices offer a suitable habitat.

### 2.2 Study species

Hyraxes were previously known as native only on the lower reaches of the eastern slopes of the Judean Mountains, with no documentation of their presence on the ridgeline area or the western slopes prior to the 1990s (Meltzer & Livneh, 1982; Mendelson & Yom‐Tov, 1988). The ridge lies at an altitude of 800–1000 m and is characterized by a moderate topography with very few natural refugia suitable for hyraxes (Ben‐Moshe & Iwamura, 2020). It probably acts as a climatic barrier as hyraxes are sensitive to cold weather and have not been found above 700 m in the study area or in other high mountainous regions of Israel (preliminary analysis of hyrax observations in the Galilee and Mt. Hermon).

Hyrax distribution has expanded significantly since 1990, perhaps as a result of their legal status as protected species and an enforced hunting ban (Mendelssohn & Yom‐Tov, 1999) but very likely also due to the increase in new habitats available to them following human land development and land‐use changes (Moran et al., 1987; Waitz et al., 2019). Their expansion pattern, as periodic observation data indicate, follows the main basins, rising from the eastern slopes towards the ridge and then down the western slopes (Figure 1). Despite the hyraxes crossing relatively similar areas in terms of topography, human density and land‐use, we identified three regions where hyrax populations exhibited different speeds of crossing and different extents of expansion from each other: populations in the north have advanced over 40 km westward compared to 4 km in the central region. In the south, no progress has been observed.

### 2.3 Datasets

#### 2.3.1 Observation data

Observation data were provided courtesy of the Israel National Parks Authority (INPA) for the years 1973–2019. Highly skilled rangers working at remote field sites conducted all the observations, which were thus not restricted only to roads or human settlements.

We used the ‘thin’ function in the r package spThin (Aiello‐Lammens et al., 2015) to reduce the biases in sampling efforts or samples of the same hyrax colony. Based on the preliminary observations (Ben‐Moshe & Iwamura, 2020), we found that hyraxes were rarely spotted at distances greater than 120 m from their shelters. Consequently, a minimum distance of 150 m between records was used to obtain 607 “thinned” observations (out of a total 1162 hyrax observations), one third and two thirds of which had occurred before and after 1991, respectively.

#### 2.3.2 Spatial dataset for environmental data

A high‐resolution (25 m) digital spatial dataset of environmental variables incorporated four main categories: geology, land‐use, climate and topography (Table 1 and Figure S2):

**TABLE 1:** Predictors used in the distribution model of the rock hyrax.

Category	Description	Retrieval information	Comment
Climate	Minimum temperature of the coldest quarter for the years 1991–2020	Retrieved from Israel Meteorological Service. Calculated as the average for the months Dec‐Feb (50 m resolution)	Used in the final model
Topography	Slope (between 0^0^–90^0^)	Survey of Israel	Used in the final model
Geology	Faults	Geological Survey of Israel, scale 1:50,000	Used in the final model
	Rock formations		Data were interpreted into “border‐potential” layer
	“Bolder‐potential” on a scale of 1–10	Converted from rock formation layers	Used in the final model
Land use	Distance from built areas and roads (highways, paved and unpaved)	Retrieved from Google Open Street Map, then corrected manually by using updated orthophotos. Distance generated in ArcMap 10. Inside human settlements only main roads were chosen	Used in the final model
	Distance from quarries	Digitized manually from orthophotos distance generated in ArcMap 10	Used in the final model. Was not used in the model for hyrax native distribution, as there were no large quarries in the area before 1991

##### Climate

To define the climatic envelope of the hyraxes, we utilized Worldclim (Fick & Hijmans, 2017) and considered a broader range of climatic variables beyond the study area alone, following Fournier et al. (2017). After examining 19 variables, we identified the minimum temperature of the coldest quarter as the biologically relevant climatic variable that affects hyrax distribution. While annual precipitation and temperature were found to be significant, they demonstrated high collinearity and were, therefore, excluded from further analysis. To obtain the relevant data for the minimum temperature of the coldest quarter in the study area, we extracted a high‐resolution (50 m) climatic layer using data from the months of December to February provided by the Israel Meteorological Service, which was used for further analysis in the study area.

##### Geology

**FIGURE 3:**
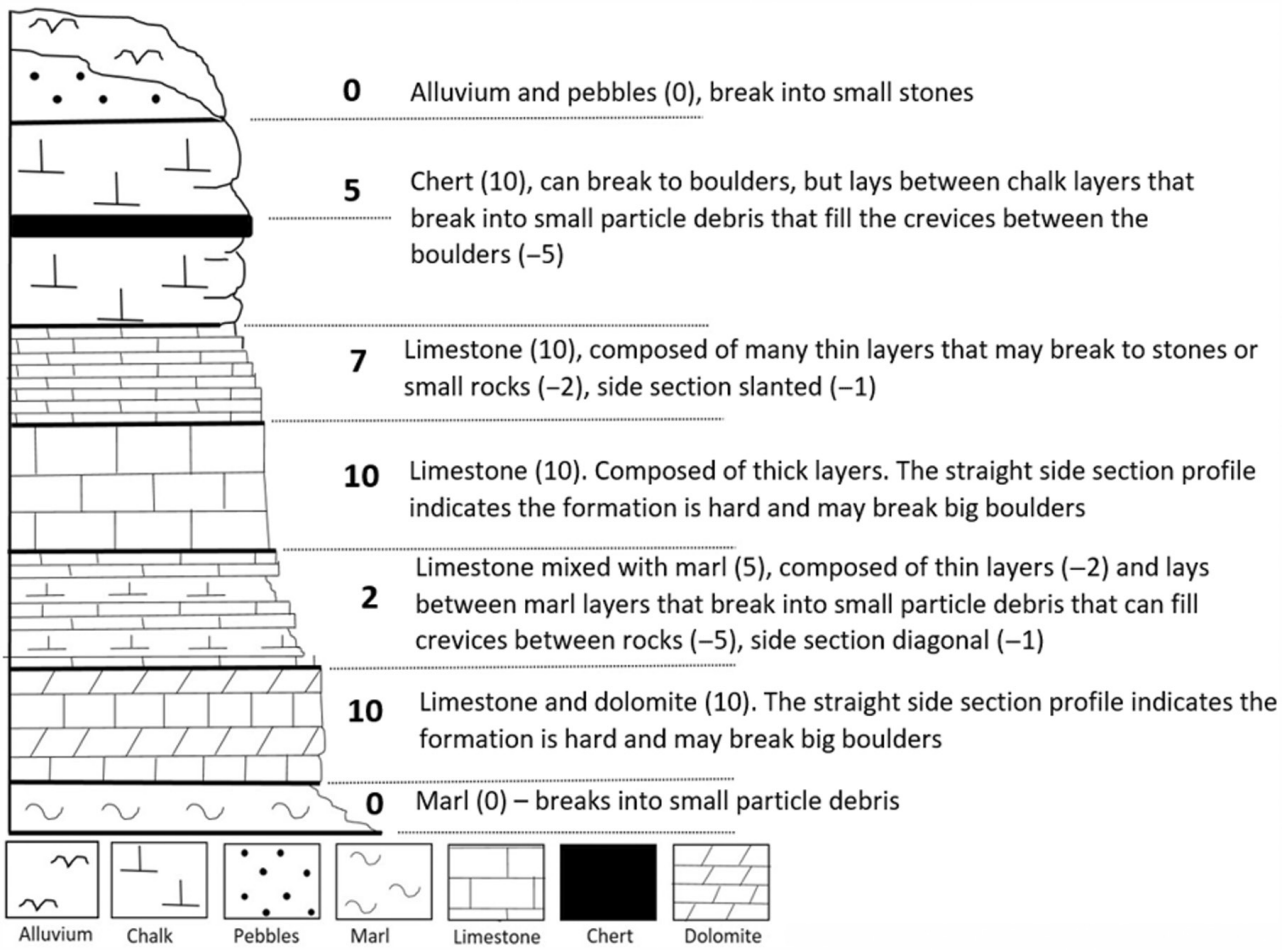
A schematic of a stratigraphic section with scores (following Table 2). Scores start by determining the main rock type in each unit, followed by reductions (marked in parentheses) based on the thickness, shape, and location of each rock type in the section. The overall score of each layer (marked in bold) cannot be lower than zero.

Following our hypothesis that geological diversity dictates the hyrax distribution patterns, we converted geological data into ecological data, which indicated the potential of each rock formation to provide a suitable habitat for the hyrax. First, we obtained high‐resolution lithological maps of the study area (Israel Geological Survey): 14 maps on a scale of 1:50,000 and two maps on a scale of 1:200,000 (where higher resolution maps were unavailable). Using the stratigraphic column for rock formations and weathering patterns developed for each stratigraphic unit, we estimated and scored the formations on each map on a 1–10 scale for their potential to break up into boulders or rocks that would be large enough to establish a suitable hyraxes habitat (i.e., >1 cubic meter [Ben‐Moshe & Iwamura, 2020])—see Figure 3. Although many of the formations appeared in multiple maps, they tended to have slightly different compositions and different weathering patterns in the different geographic locations within the region. Consequently, a scoring system applied to each map separately provided a better method for identifying potential areas suitable for a hyrax habitat (Table 2 and Figure 3).

**TABLE 2:** Scores for rock units based on their ability to break into large boulders with crevices – the rock hyrax’ preferred habitat.

Rock characteristics	Description	Score
Type	Hard rocks—Limestone, dolomite and flint	10
	Conglomerate	5
	Soft rocks—Alluvium, marl and chalk	0
	Mixed soft and hard rocks	5
Durability by shape in stratigraphic section	Straight section edge	0
	Slanted section edge	−1
	“Eaten” section edge	−2
Thickness	Formation thickness <10 m	−2
	Formation comprised of thin layers	−2
Location between other rock layers	lays between soft layers that break into small particle debris that can fill crevices	−5

The parameters we used for scoring were durability and the thickness of the specific formation (greater durability and thickness receive higher scores). A lower score was given for formations mixed with soft lithology such as marl or chalk, which disintegrate into small grains that can fill the crevices between the rocks and thus limit the cavity space. Chert and dolomite are considered durable rocks and received a high score. We then converted the geological map into a “potential‐boulder” map and merged the 16 maps, based on the scores of the formations, into one map covering the entire study area. To validate our scoring approach for identifying “rock potential”, we located 135 sites on an orthophoto basis, where we identified dirt/rock debris near four different main roads (routes 5, 1, 449 and 317, north‐to‐south) carved into the mountainside. Of these sites, 45 were randomly selected then inspected in the field and rated on a scale of 1–4 for their suitability for hyrax habitats (based on Ben‐Moshe & Iwamura, 2020; Salah et al., 2020, Table S2). Using a cumulative link model (Christensen, 2011), we tested for correlation between the lithological score predicted and the habitat suitability measure observed.

The other variables used for the distribution model of the rock hyrax (i.e., land‐use, topography and geological faults) are described in Table 1.

All layers were up‐scaled to a 25 m resolution.

### 2.4 Species distribution model

We used the “Maximum Entropy” model produced by the free MaxEnt V4.0.2 (Phillips et al., 2006; Phillips & Dudík, 2008) software, as this is one of the most effective models for predicting species distribution on the basis of presence‐only data (Elith et al., 2011). The algorithm calculates the most probable potential geographic distribution of a species, based on the relationship between the geographical data and the known distribution of the target species (Elith et al., 2011). The parameter settings for the MaxEnt model were set according to the MaxEnt Model v. 3.3.3e Tutorial (Phillips, 2010). The performances of different models were then evaluated using the R package “ENMeval” version 0.2.0 (Kass et al., 2021), with the best‐fitting model being the one with the lowest AICc (small‐sample corrected Akaike Information Criterion [Burnham & Anderson, 2004]) score. The parameters with the best fit were employed in MaxEnt to predict suitable areas for the hyrax expansion across the Judean Mountains. To estimate the contribution of the environmental and anthropogenic factors to the various stages of the hyrax expansion, we ran the model using different spatial extents and occurrence records: (A) historical range with native population records (until 1991); (B) historical range with “new” records (1991 onwards); and (C) entire study range with all records (full model).

The accuracy of each model prediction was quantified using two of the most frequently used measures, i.e., the Receiver Operating Characteristic (ROC) curve (AUC) and the True Skill Statistic (TSS), both are independent of prevalence (Allouche et al., 2006). The AUC is a threshold‐independent measure and ranges from 0.5 for an uninformative model to 1 for perfect discrimination (Franklin & Miller, 2013), while TSS is evaluated based on a selected threshold and ranges from −1 to +1, where +1 indicates perfect agreement and values of zero or less indicate a performance no better than random (Allouche et al., 2006). However, as both indices were criticized for their reliability in measuring the performance of models based on presence‐only data (Lobo et al., 2008), we also employed the Boyce index which is probably more appropriate for such models (Hirzel et al., 2006). It is continuous and varies between −1 and + 1, where positive values indicate that model predictions are consistent with the distribution of presences in the evaluation dataset and near‐zero values implies that the model outputs are not different from random. Negative values indicate counter predictions.

### 2.5 Assessment of land‐use change and geology interaction in facilitating hyrax distribution

To assess the role of interactions between geology and land‐use in the distribution of the rock hyrax, we divided the study area into four combination categories based on the proximity to the land‐uses that create boulders (i.e., roads and built areas) and the local geological potential (Table S3): (a) Close to human land‐uses and high‐geological score; (b) Close to human land‐uses but low‐geological score; (c) Far from human land‐use and high‐geological score; and (d) Far from human land‐use and low‐geological score.

The number of hyrax records in each combination was then compared to the expected number of observations if hyraxes were evenly distributed using the chi square test.

### 2.6 Analysis of the natural and the anthropogenic factors that create hyrax habitats, using high‐resolution orthophotos

To determine the factors contributing to the creation of hyrax habitats along their expansion course (i.e., eastern slopes, ridgeline and western slopes), we visually analyzed high‐resolution (12.5 cm/pixel) orthophotos of the locations of the hyrax observations. As rocks suitable for hyraxes are large (>1 cubic meter [Meltzer & Livneh, 1982]), rock piles could be easily identified at such resolution (Figure S1). We matched each observation location to the nearest rock pile observed at a distance of up to 150 m. By visually analyzing the orthophotos, we were able to determine the origin of the rock pile according to the following categories: “natural”, “road”, “built area” and “quarry”. If no rock pile was observed within this distance, or its origin could not be identified, we marked it as “unidentified origin”.

## 3 RESULTS

### 3.1 In‐situ validation of the “boulder‐potential” score

We found a significant, strong correlation (*p* < .001) between the scores given to lithological layers based on their potential to break into large boulders and the habitat suitability score for hyraxes of the debris formed along the roads surveyed (Table S2).

### 3.2 Model performance and variable importance

MaxEnt models' outputs for predicting hyrax distributions are shown in Table 3. While geology is a primary influencer in all models, the relative contribution of other factors to the models varies along the hyrax expansion stages. Although environmental factors (climate, slope and distance from faults) played an important role in the model of native hyrax populations (Column A), land‐use shows the greatest importance in the model of recent hyrax observations in the same area (Column B).

**TABLE 3:** Percent contribution and permutation importance of the predictor variables for the MAXENT distribution models for the rock hyrax.

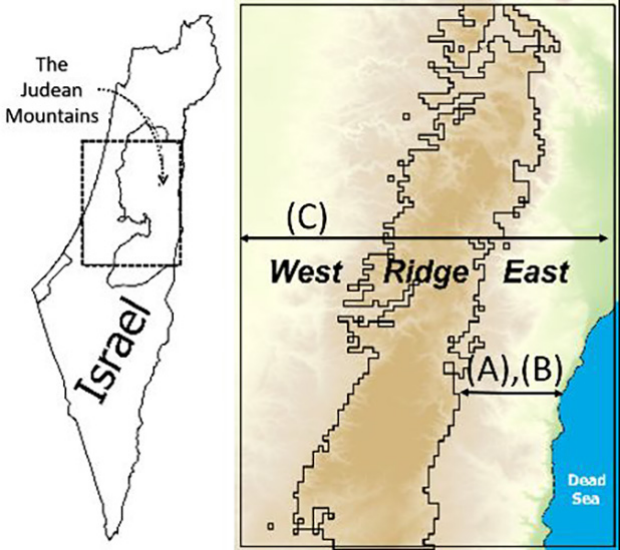		(A) SDM of the native population in the historic range—Eastern slopes below 700 m (observations prior to expansion in 1991)				(B) SDM based on new records (1991–2019) in the historic range—Eastern slopes below 700 m				(C) SDM in all the study extent (observations across the Judean Mountains 1973–2019)			
		AUC	TSS	AICc	Boyce	AUC	TSS	AICc	Boyce	AUC	TSS	AICc	Boyce
		0.87	0.61	6138.2	0.96	0.89	0.64	4564.8	0.99	0.91	0.67	16,230.9	0.99
		Permutation importance				Permutation importance				Permutation importance			
Climate	Mean temperature of coldest quarter	15.8%				2.8%				**23.6%**			
Geology	Lithology	**44.1%**				**40.5%**				**32.9%**			
Natural weathering forces	Slope	14.3%				6.7%				6.1%			
	Geological faults (distance from)	10.5%				2.2%				9.4%			
Land‐use	Roads, built area, quarries (distance from)	15.3%				**47.8%**				**28.0%**			

*Note*: Values of 20% percent contribution and higher are highlighted.

The results from the full model that includes the whole study area and both native and expanding hyrax observations (Column C) indicate that geology, climate and land‐use, together account for around 90% of the hyrax distribution pattern, with each having a relatively similar contribution.

### 3.3 Distribution of potential suitable and unsuitable areas for hyraxes

**FIGURE 4:**
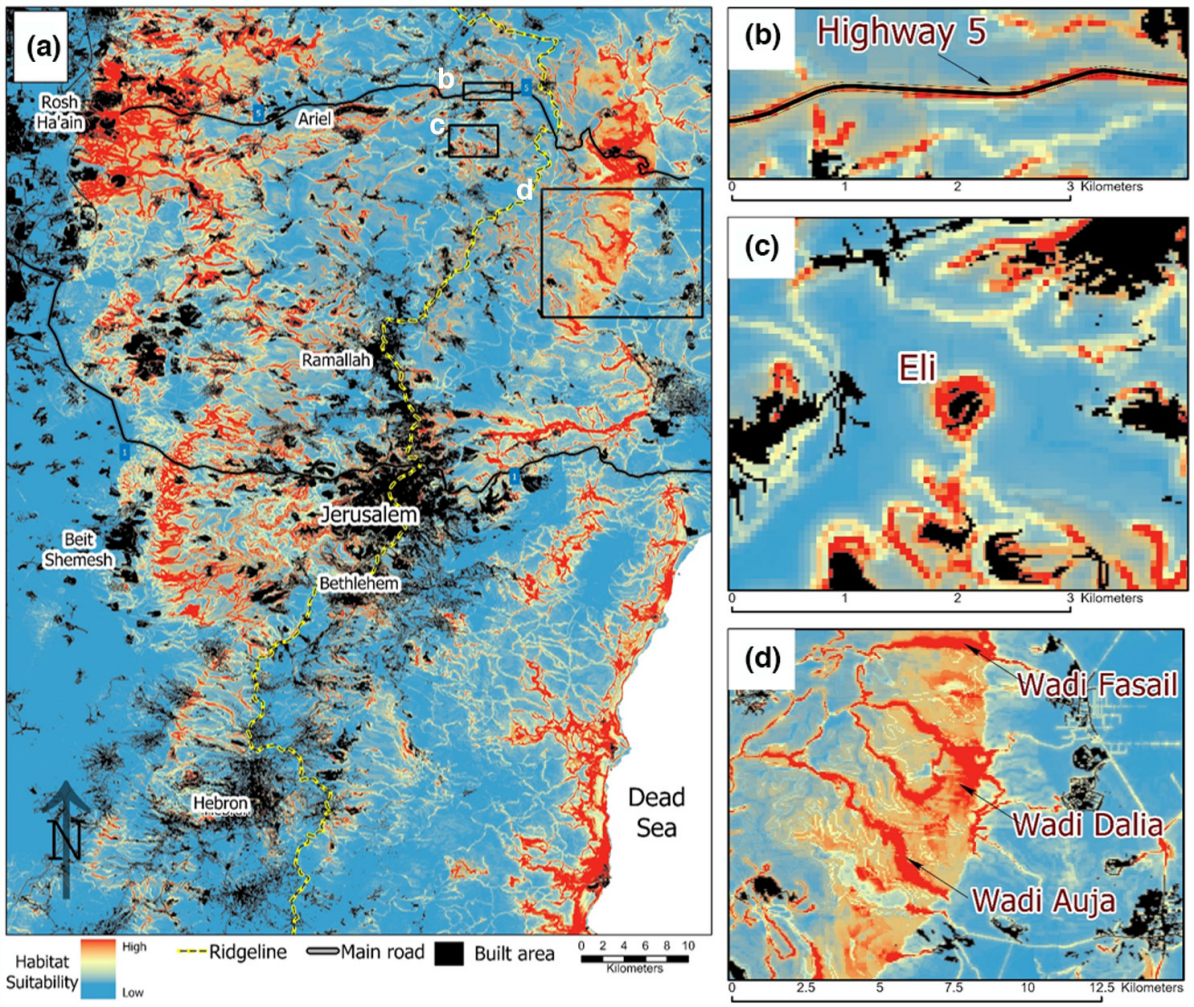
Probabilistic distribution maps of the rock hyrax in the Judean Mountains. (a) Overall the rock hyrax distribution estimates in the Judean Mountains. Colors represent predicted habitat suitability: from blue—low suitability, to red—high suitability. The map enables detecting land use types and geomorphological features facilitating hyrax expansion: (b) Potential movement corridors along roadsides. (c) Habitat patches around human settlements near the ridgeline. (d) Potential movement Corridors along canyons in the eastern slopes.

The probabilistic distribution map of the full model with the marking of built‐up areas (Figure 4) enabled the detection of four factors that can explain the hyrax dispersal patterns and the differences in expansion between the northern, central and southern populations: (a) regions with suitable conditions on the eastern slopes, such as canyons and cliffs, are closer to the ridgeline in the north and central areas, but far from the ridgeline in the south; (b) regions with suitable conditions for hyrax settlement on the western slopes are large in the north and the center but small in the south; (c) possible movement corridors along roads and human settlements, which enable hyrax dispersion across the ridgeline in otherwise unsuitable environmental conditions, are found in the north and the center; and (d) large, continuous urban areas that hinder hyrax movement in the central and southern populations, but not in the north. To cross these urban areas, rock hyraxes had first to adjust to the urban environment (Ben‐Moshe & Iwamura, 2020).

The combined effects of these factors indicate that the rock hyrax northern populations had more favorable conditions for expansion than the central and southern populations. The central population crossed the urban area of Jerusalem in a bottleneck after initially settling in urban sites (Ben‐Moshe & Iwamura, 2020). Observation data indicate that the hyraxes crossed this bottleneck to reach the western slopes (first observations on the western slopes in 2009), where their population is now expanding and has the potential to expand further westwards, where large suitable areas are still uninhabited by hyraxes.

### 3.4 Geology–land‐use interaction effect on hyrax distribution

Table 4 presents chi square test values for the hyrax records in relation to the potential of the local geological units to break into boulders and the distance from human land‐uses, which are associated with creating debris (roads and built areas). According to these results, hyraxes are significantly distributed in areas where the local geology supports the creation of rock piles formation. However, while hyrax records were mostly far from roads and built areas prior to the 1990s, new records are largely located in areas where the geological potential intersects with these land‐use practices.

**TABLE 4:** Chi Square test results for assessing land‐use geology interaction on hyrax distribution.

	Standardized residuals				
	Good geology land‐use far	Good geology land‐use near	Bad geology land‐use near	Bad geology land‐use far	*p*‐Value
East Native (observations 1973–1990)	**10.809479**	6.838672	−3.034163	−9.90958	<.01
East new (observations after 1990)	5.782782	**26.871947**	−3.587549	−16.827981	<.01
West (expanding, observations after 1991)	−2.187066	**17.809674**	−8.228813	−6.149157	<.01

*Note*: Categories that were the furthest from even distribution are highlighted.

### 3.5 Origin of the hyrax shelters

**FIGURE 5:**
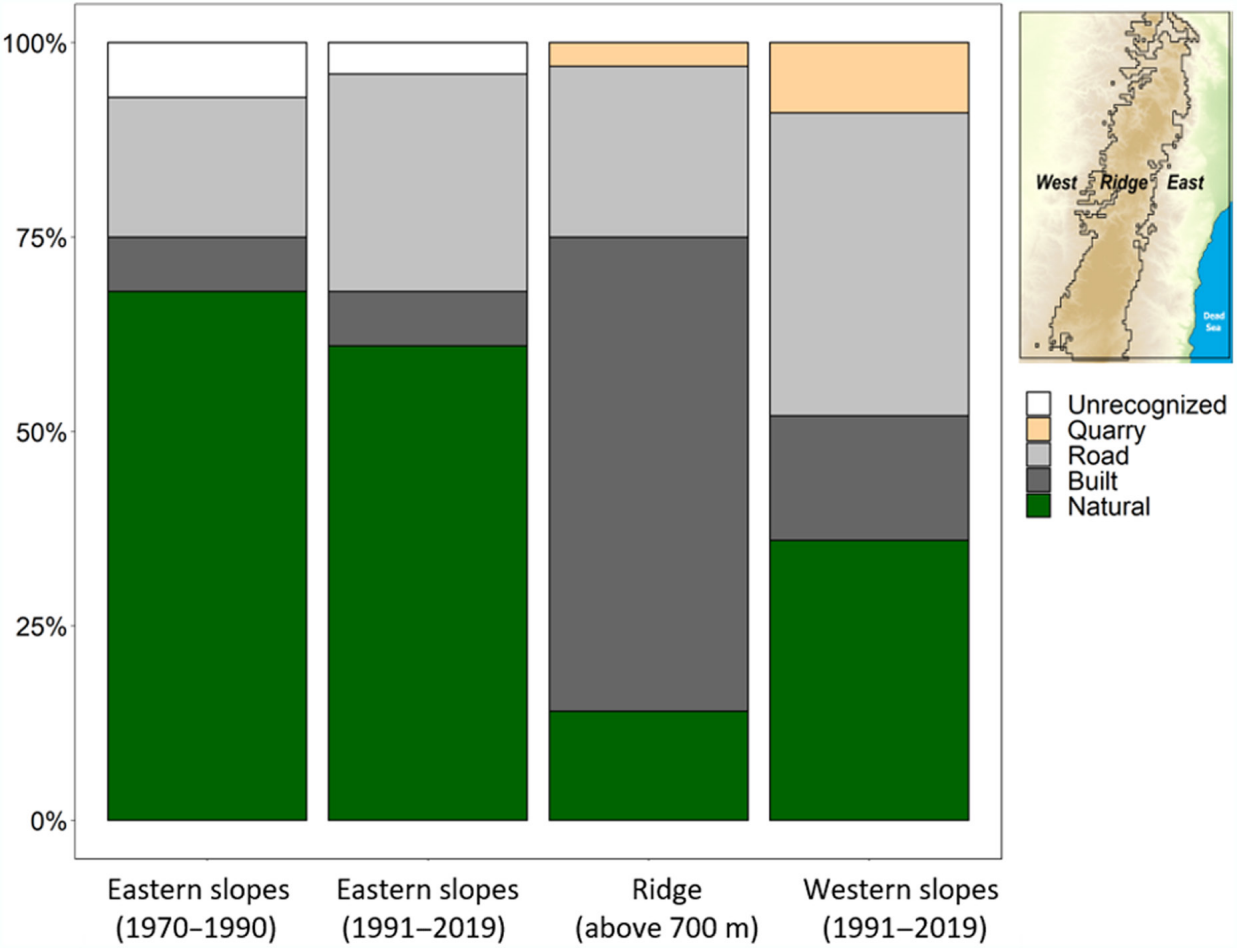
Inhabited rock mounds by their forming cause in the study regions. In their native range (eastern slopes), hyraxes used mostly natural habitats. In the expansion areas (i.e., the ridge and western slopes), hyraxes used mostly rock piles created by human activity. In the higher regions near the ridgeline, hyraxes were concentrated around human settlements. In the western slopes, hyraxes used rock piles along roads, near settlements and quarries, while only a third of the expanding populations used natural mounds.

Analysis of hyrax habitats from orthophotos (Figure 5) indicates that the expansion of hyraxes across the Judean Mountains is facilitated by land‐use change‐induced habitats, as also revealed in the outputs of the SDMs. Native hyrax populations on the eastern slopes, as observed until 1990, inhabited mostly natural habitats (68%), followed by roadsides (18%) and built‐up areas (7%). After 1990, although the hyraxes in their historical range still relied mostly on natural habitats (61% of total habitats), they also begun to exploit more rock mounds along roads (28%).

However, these expanding populations relied on rock piles formed by land‐use change activities (86% and 64% in the expansion regions of the ridgeline and western slopes, respectively). On the ridgeline, most hyraxes inhabited rock mounds in human settlements (63%), followed by roadsides (22%) and quarries (3%). On the western slopes of the expansion front, natural habitats represented only 36%, while anthropogenic habitats, such as roads, provided the majority of dwellings (39%), followed by human settlements (19%) and quarries (9%).

## 4 DISCUSSION

**FIGURE 6:**
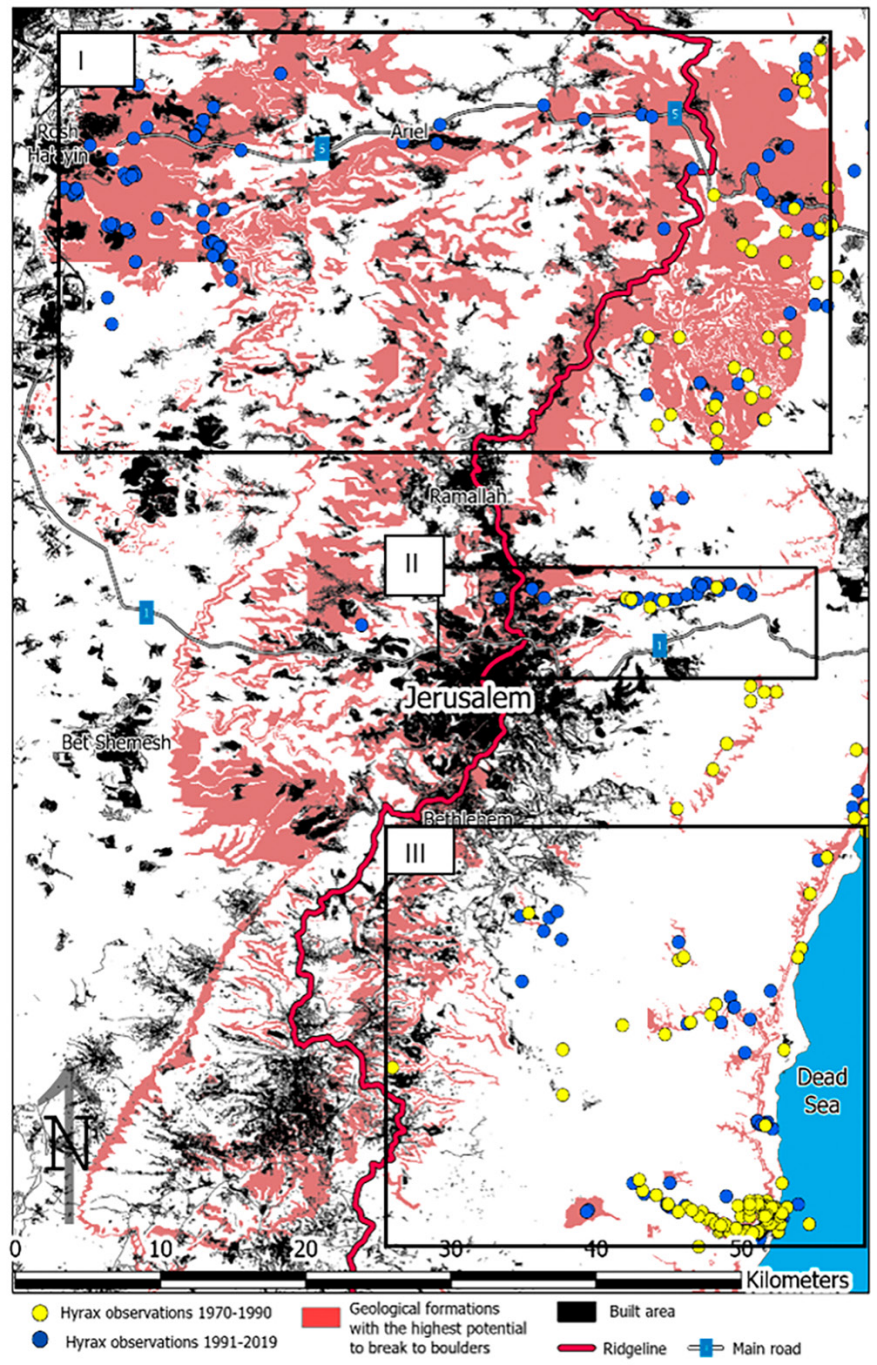
Rock hyrax expansion explained through geological formations, roads and built areas. (Native population is indicated as yellow circle, new populations are in blue. Rock formation with highest potential [Scores 9 and 10 in Table 2] are shown in pink polygons). **I.** The North population progressed across the ridgeline where hard rock formations weathered by human land use (such as Highway 5) created continuous passage of rock mounds. Numerous new populations settle on the low western slopes where the climate is warmer than the ridge, and there is a combination of hard rock formations and human development. **II.** Central population – is native at Wadi Qelt canyon carved through hard rock formations. Hyrax expansion to the urban areas is through construction on hard formations that created new rock mounds. Continuous urban area that stretches for 30 km (from Bethlehem through Jerusalem till Ramallah) creates an obstacle for further progression westwards. **III.** South population does not have continuous passage because continuously built areas on the ridgeline (Hebron) and limited areas suitable for colonization on the western slopes.

Geology, along with climate, is one of the most fundamental forces behind the distribution of plants and animals (Antonelli et al., 2018; Gillspie & Roderick, 2014; Kruckeberg, 2002; Moriarty & Honnery, 2004; Ott, 2020). In the Anthropocene epoch, however, land‐use changes have become the most influential factor in altering species' distribution (Baillie et al., 2004; Sala et al., 2000). While numerous studies have demonstrated that land‐use changes alone do not always modify species distributions but rather within the constraints or interaction with climate (Améztegui et al., 2010; Cannizzo Id & Griffen, 2019; Peters et al., 2019) and climatic changes (Guo et al., 2018; Hof et al., 2011; Jetz et al., 2007; Mantyka‐Pringle et al., 2015; Manzoor et al., 2021; Oliver & Morecroft, 2014), there is a dearth of studies that explicitly demonstrate such effects of land‐use change‐geology interactions. Our findings suggest that the interaction between geology and land‐use significantly influenced the recent distribution of the rock hyrax across the Judean Mountains by producing large boulders, which serve as new habitats and stepping‐stones that enable the animals to traverse climatic barriers and expand geographically (Figure 6).

The range expansion of native wildlife by roads and built‐up areas, as found in our study, contrasts other negative anthropogenic impacts such as habitat loss and fragmentation (Forman et al., 2003). Roads in particular are considered among the types of land‐uses presenting the highest risk to wildlife due to vehicle collision (Coffin, 2007; Forman et al., 2003; Magioli et al., 2019), and by setting both physical and behavioral barriers to animal movement (Forman & Alexander, 1998; McGregor et al., 2008; Shepard et al., 2008). In the Judean Mountains, roads that cut across hard rock formations create large rock mounds on the sides providing a continuous shelter network for the hyraxes and thus facilitating their movement (Figure 2). Road construction often exposes the local bedrock and soil and provides habitats that differ from their adjacent areas, and these might support the dispersal of native species across inferior environments. For example, grasses growing on exposed soils along roadsides support the dispersion of grassland rodent species in both forests and extensive agricultural areas (Getz et al., 1978); and the common wall lizard (*Podarcis muralis*), for example, has expanded to colder regions in Romania by sheltering at stony roadsides (Gherghel et al., 2009). It is also commonly recognized that invasive plants too are able to spread in the disturbed soils along roadsides (Follak et al., 2018; Lázaro‐Lobo & Ervin, 2019; McDougall et al., 2018).

Human settlements affect hyrax expansion both negatively and positively. Hyraxes settling close to human settlements also potentially benefit from higher ambient temperatures during the winter (Pickett et al., 2001), and enjoy year‐long foraging grounds in residential parks and gardens (Ben‐Moshe & Iwamura, 2020; Mendelssohn & Yom‐Tov, 1999; Naylor, 2015). However, areas of intense human settlement, such as large urban areas, hinder the movement of wildlife (Braaker et al., 2014; Tannier et al., 2016) and impede hyrax expansion. In the central Judean Mountains, a continuous urban sprawl stretches from Bethlehem to Jerusalem and Ramallah, perpendicular to the dispersion of the hyraxes across the mountains (Figure 6). To cross such an alien environment, the hyraxes had first to adapt to urban areas, use urban shelters as stepping‐stones, and eventually cross the city along its narrowest part (Ben‐Moshe & Iwamura, 2020). Such a process takes time, and while the observation data indicate that hyraxes first appeared near Jerusalem in the 1990s, the first recorded sightings on the western side of the city only occurred in 2009. The northern populations did not face such urban obstacles during their dispersal and thus expanded much faster onto the western slopes.

Land‐use change is considered to be one of the major drivers of infectious diseases (Daszak et al., 2000; Patz et al., 2004). The expansion of wildlife species into human‐populated areas has been reported to cause the emergence of zoonotic infectious diseases (Bradley & Altizer, 2006; Murray et al., 2016; Patz et al., 2004; Soulsbury & White, 2015). In both the Palestinian Territories and Israel, the rock hyrax is an important host of *Leishmania tropica*, which causes severe cutaneous leishmaniasis (Jaffe et al., 2004; Talmi‐Frank et al., 2010). Understanding the key drivers of rock hyrax expansion is thus of critical concern in order to mitigate the negative health impacts. Our findings emphasize how the interaction between geological substrates and land‐use (e.g., roads) creates corridors and stepping stones for the hyrax across previously unsuitable regions for their expansion. This information can assist health and environmental workers to locate those high‐quality patches favorable to the hyraxes (Figure 4), and assess their potential future expansion directions and bottlenecks (Figure 4b–d). For example, compared to the overall Judean Mountains, the area featuring suitable geological properties to support hyrax expansion is limited (Figure 6). Consequently, the efforts and resources spent on preventing the creation of new such piles during construction and pavement works can be better focused.

The interaction of geology and land‐use change and its impacts on ecological processes have been discussed previously (Jones & Faheem, 2020). For example, carbon emissions from the Amazon constitute a cumulative process resulting from geological substrate and land‐use interaction (Asner et al., 2010). It has also been found that microbial carbon consumption is affected by the interaction between geological substrates and land‐use (Zheng et al., 2019). The consistency of the rock substrate and fertility of the soil determine which areas will be plowed, while the abruptness of the relief determines the possibilities for grazing or forest use (García‐Quintana et al., 2004), further suggesting that the interplay between geology and land‐use plays a key role in sustainable soil management (Haygarth & Ritz, 2009). Biological knowledge, such as of plant assemblages, is used in interpreting geological phenomena such as groundwater surveys, mapping of soil properties, and in mineral prospecting (Kruckeberg, 2002; Wei et al., 2020), while botanists use geological maps to understand the distribution of local flora (Antonelli et al., 2018; du Puy & Moat, 1996). Similarly, geological knowledge can be of use in studies engaging with the distribution of wildlife with a strong affinity to geomorphology (e.g., bats in caves and crevices) or geochemistry (e.g., amphibians in water bodies). In this study, we have demonstrated how the construction of roads and infrastructure, without considering the geological‑ecological interactions, has facilitated the expansion of a wildlife species that can spread an infectious disease. Understanding other particular geological qualities and their interaction with land‐use change, such as a soil's ability to retain moisture, could assist in detecting irrigated areas susceptible to host‐pest species such as sandflies (Feliciangeli, 2004) or mosquitoes (Norris, 2004). Considering that geological factors and land‐use processes are highly connected in their effect on ecological systems, it is crucial to integrate geological data with ecological knowledge to ensure sustainable land‐use planning.

## AUTHOR CONTRIBUTIONS

**Noam Ben‐Moshe:** Conceptualization (equal); data curation (lead); formal analysis (equal); investigation (lead); methodology (equal); validation (equal); visualization (equal); writing – original draft (equal); writing – review and editing (equal). **Marcelo Rosensaft:** Data curation (supporting). **Takuya Iwamura:** Conceptualization (equal); data curation (supporting); formal analysis (equal); investigation (supporting); methodology (equal); validation (equal); visualization (equal); writing – original draft (equal); writing – review and editing (equal).

## ACKNOWLEDGEMENTS

The authors would like to express their sincere gratitude to Lior Enmar for his invaluable contribution in translating geological data into ecological information. We would also like to thank Dr. Yoav Avni from the Israel Geological Survey and Perach Nuriel from the University of Geneva for their insightful comments and suggestions, which significantly improved the quality of this research.

## Supporting information


Appendix S1.

## Data Availability

The data that support the findings of this study are openly available in Dryad at https://datadryad.org/stash/share/ugYbiSqYEo82z5mWcQHdlpNI9XtFsvsjjJP121TbFVk.
