# Supplementary material for: Land‐use changes interact with geology to facilitate dispersal of the rock hyrax (Procavia capensis) and leishmaniasis across Israel and the West Bank
Source: Ecol Evol. 2023 Mar 21;13(3):e9915. doi: 10.1002/ece3.9915 (PMC10030234; doi:10.1002/ece3.9915)
Supplement: Supplementary file 1 — Appendix S1. [file ECE3-13-e9915-s001.docx]

**Appendix**

**Fig S1:** Hyrax observations in rock piles by land-use as analyzed in high-resolution (12.5 cm/pixel) orthophotos:

The observations are marked as yellow circles, area with rock piles by white borders (digitized manually).

1. Natural boulders in a canyon (Wadi Qelt).
2. Artificial rock piles formed by roads (north Judean Mountains),
3. Artificial rock piles under human settlement (Jerusalem).
4. Artificial rock piles in a deserted quarry (Jerusalem).


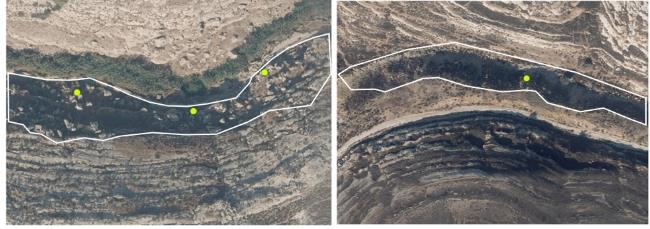

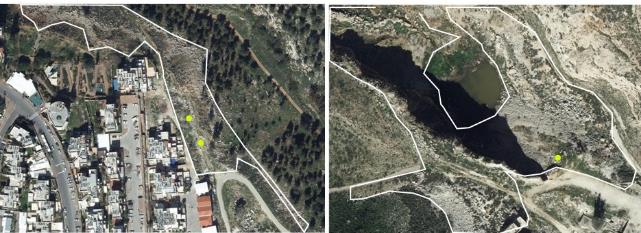


(A)

(B)

(C)

(D)

**Road**

**Debris from road**

**Quarry**

**Debris from built area**

**Built area**

**Fig S2:** Maps of the predictors used in the SDM: a) Geology (Scores if rock ability to break into large boulders); b) Slope; c) Distance from nearest geological fault; d) Mean temperature of coldest quarter; e) Distance from nearest roads and built area; f) Distance from nearest quarry

| 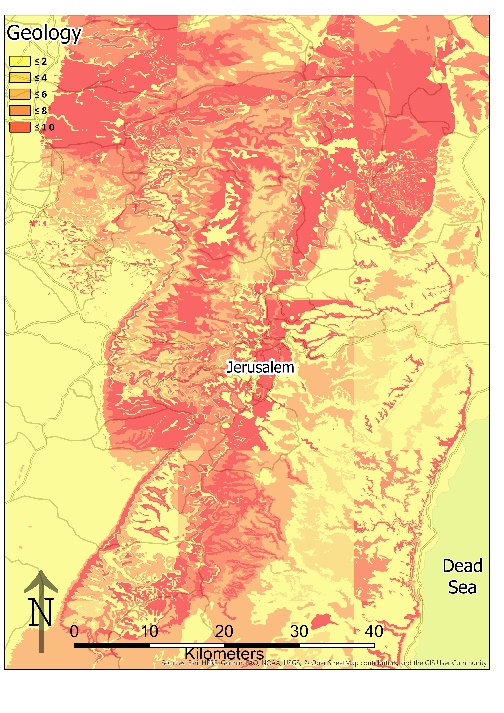a) | 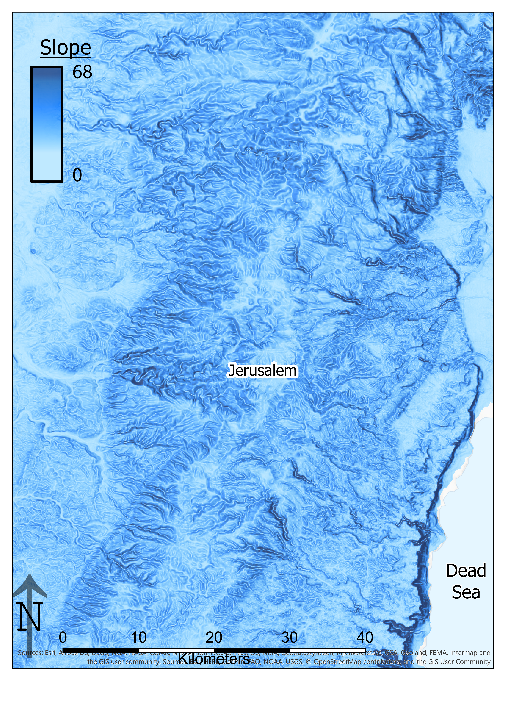b) | 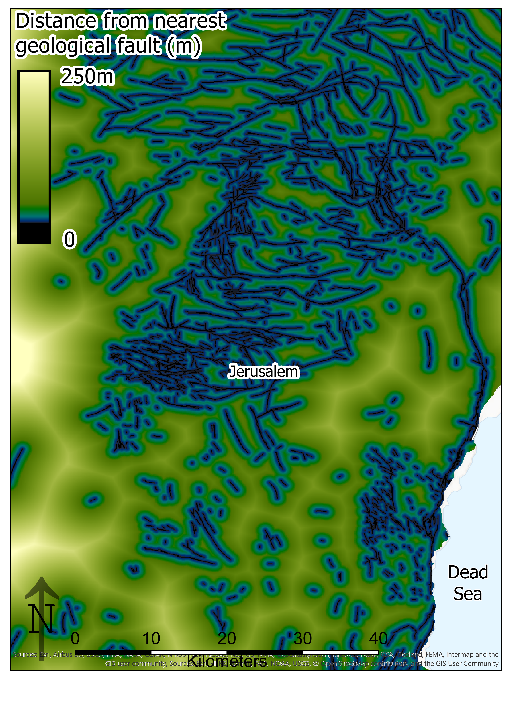c) |
| --- | --- | --- |
| 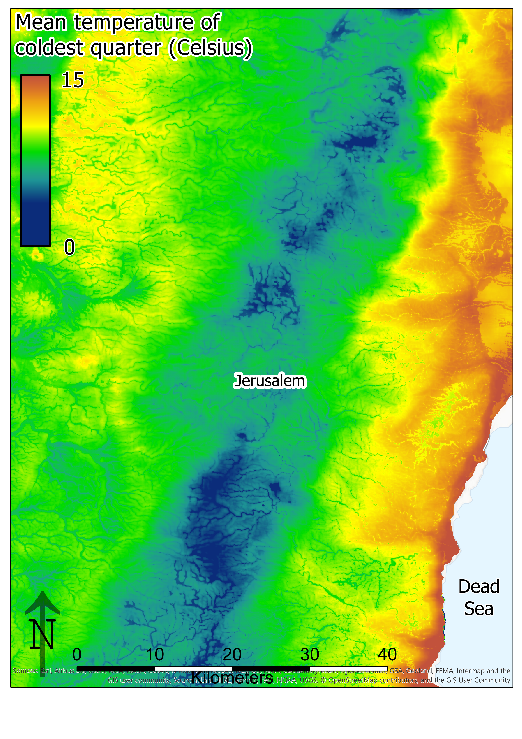d) | 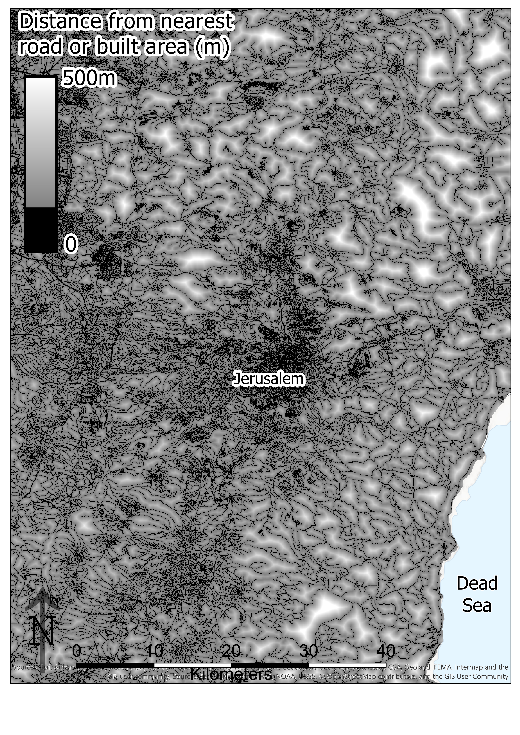e) | 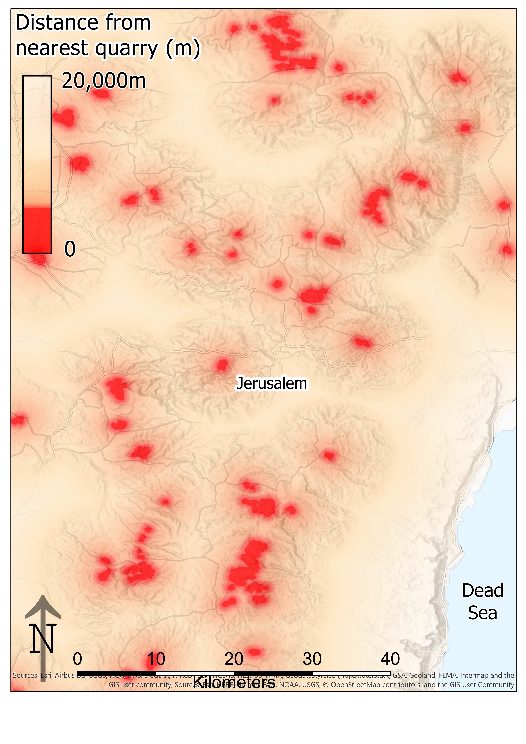f) |

**Table S1**: Survey sites used for validating the scores given to geological units by their predicted potential to break into large boulders and create suitable habitats for rock hyraxes

| **ID** | **Long** | **Lat** | **Road Number** | **Geological unit** | **Boulder-potenyial score (predicted)** | **Habitat suitability score found** |
| --- | --- | --- | --- | --- | --- | --- |
| 1 | 35.212 | 32.123 | 5 | Veradim | 10 | 4 |
| 2 | 35.202 | 32.121 | 5 | Veradim | 10 | 4 |
| 3 | 35.191 | 32.118 | 5 | Veradim | 10 | 4 |
| 4 | 35.217 | 32.124 | 5 | Veradim | 10 | 3 |
| 5 | 35.218 | 32.125 | 5 | Veradim | 10 | 4 |
| 6 | 35.224 | 32.125 | 5 | Veradim | 10 | 3 |
| 7 | 35.233 | 32.126 | 5 | Bina | 10 | 4 |
| 8 | 35.252 | 32.124 | 5 | Bina | 10 | 3 |
| 9 | 35.267 | 32.116 | 5 | Aminadav | 9 | 4 |
| 10 | 35.276 | 32.118 | 5 | Aminadav | 9 | 4 |
| 11 | 35.282 | 32.12 | 5 | Aminadav | 9 | 4 |
| 12 | 35.289 | 32.119 | 5 | Aminadav | 9 | 4 |
| 13 | 35.301 | 32.121 | 5 | Aminadav | 9 | 3 |
| 14 | 35.31 | 32.12 | 5 | Aminadav | 9 | 2 |
| 15 | 35.335 | 32.115 | 5 | Dir Hana | 8 | 3 |
| 16 | 35.349 | 32.09 | 5 | Sakhnin | 8 | 4 |
| 17 | 35.351 | 32.085 | 5 | Sakhnin | 8 | 1 |
| 18 | 35.355 | 32.073 | 5 | Dir Hana | 8 | 1 |
| 19 | 35.366 | 32.072 | 5 | Dir Hana | 8 | 2 |
| 20 | 35.354 | 32.073 | 5 | Dir Hana | 8 | 4 |
| 21 | 35.400 | 32.066 | 5 | Dir Hana | 8 | 4 |
| 22 | 35.406 | 32.067 | 5 | Dir Hana | 8 | 4 |
| 23 | 35.429 | 32.064 | 5 | Sakhnin | 8 | 4 |

| **ID** | **Long** | **Lat** | **Road Number** | **Formation** | **Score_pred** | **Score_foun** |
| --- | --- | --- | --- | --- | --- | --- |
| 24 | 35.424 | 32.059 | 5 | Dir Hana | 8 | 4 |
| 25 | 35.430 | 32.052 | 5 | Dir Hana | 8 | 3 |
| 26 | 35.276 | 31.788 | 1 | Menuha | 0 | 1 |
| 27 | 35.281 | 31.785 | 1 | Menuha | 0 | 1 |
| 28 | 35.302 | 31.798 | 1 | Menuha | 0 | 1 |
| 29 | 35.333 | 31.808 | 1 | Meshash | 4 | 1 |
| 30 | 35.347 | 31.814 | 1 | Meshash | 4 | 1 |
| 31 | 35.389 | 31.817 | 1 | Meshash | 4 | 1 |
| 32 | 35.398 | 31.807 | 1 | Menuha | 0 | 1 |
| 33 | 35.409 | 31.800 | 1 | Menuha | 0 | 1 |
| 34 | 35.427 | 31.797 | 1 | Menuha | 0 | 1 |
| 35 | 35.141 | 31.410 | 317 | Bina Drorim | 5 | 3 |
| 36 | 35.127 | 31.399 | 317 | Bina Drorim | 5 | 1 |
| 37 | 35.109 | 31.384 | 317 | Bina Drorim | 5 | 2 |
| 38 | 35.177 | 31.440 | 317 | Bina Drorim | 5 | 1 |
| 39 | 35.101 | 31.375 | 317 | Bina Drorim | 5 | 2 |
| 40 | 35.066 | 31.371 | 317 | Bina | 5 | 2 |
| 41 | 35.356 | 31.914 | 449 | Timrat | 10 | 3 |
| 42 | 35.354 | 31.913 | 449 | Timrat | 10 | 4 |
| 43 | 35.350 | 31.914 | 449 | Timrat | 10 | 3 |
| 44 | 35.376 | 31.912 | 449 | Menuha | 0 | 1 |
| 45 | 35.378 | 31.912 | 449 | Menuha | 5 | 2 |

‘**Long’, ‘Lat’** – The coordinates of the survey point in WGS40

**‘Road num’** – Road number

**‘Formation’** – The Hebrew name of the geological unit that constitutes the debris.

**‘Score_pred’** – "Boulder potential score" assigned to the geological unit in the location. Ranges from 0 (no potential), to 10 (highest potential for boulders with crevices between them)

**‘Score_foun’** – The actual estimation of habitat suitability of the debris as habitat for the hyraxes. Ranges from 1 (not suitable) to 4 (very suitable).

**Table S2**: Results of a cumulative link model for the correlation between the geological units predicted potential to break into large boulders and the observed habitat suitability

 link  threshold nobs logLik AIC   niter max.grad cond.H
 logit flexible  45   -39.90 87.81 5(0)  3.80e-11 3.2e+03

Coefficients:
           Estimate Std. Error z value Pr(>|z|)    
Score_pred   0.7759     0.1730   4.486 7.27e-06 ***
---
Signif. codes:  0 ‘***’ 0.001 ‘**’ 0.01 ‘*’ 0.05 ‘.’ 0.1 ‘ ’ 1

Threshold coefficients:
    Estimate Std. Error z value
1|2    3.827      1.156   3.312
2|3    5.248      1.340   3.917
3|4    6.800      1.503   4.524

**Table S3**: Hyrax observations (thinned) in the study area by different land use-geology combinations:

“Good geology” = areas were local geological units were classified as having high score (>8) for their potential to break into boulders.

“Bad geology” = areas were local geological units were classified as having a score (≥8) for their potential to break into boulders.

“Near land-use” = areas in distance <150 m from roads/built areas

“Far land-use” = areas in distance ≥150 m from roads/built areas

| **Eastern mountains (Native distribution, records 1973-1990)** | Good geology far land-use | Good geology near land-use | Bad geology near land-use | Bad geology far land-use | **Total** |
| --- | --- | --- | --- | --- | --- |
| **Area (sqkm)** | 407.8 | 191 | 394 | 1246 | **2238.8** |
| %total area | 0.182 | 0.085 | 0.176 | 0.557 | 100% |
| **Observations (thinned)** | 46 | 18 | 14 | 19 | **97** |
| %total observations | 47% | 19% | 14% | 20% | 100% |

| **Eastern mountains (Native distribution, records 1991-2019)** | Good geology far land-use | Good geology near land-use | Bad geology near land-use | Bad geology far land-use | **Total** |
| --- | --- | --- | --- | --- | --- |
| **Area (sqkm)** | 407.8 | 191 | 394 | 1246 | **2238.8** |
| %total area | 0.182 | 0.085 | 0.176 | 0.557 | 1.000 |
| **Observations (thinned)** | 93 | 155 | 29 | 22 | **299** |
| %total observations | 31% | 52% | 10% | 7% | 100% |

| **Western mountains (expanding distribution records 1991-2019)** | Good geology far land-use | Good geology near land-use | Bad geology near land-use | Bad geology far land-use | **Total** |
| --- | --- | --- | --- | --- | --- |
| **Area (sqkm)** | 333.2 | 594.7 | 1669 | 491.9 | **3088.9** |
| %total area | 0.108 | 0.193 | 0.540 | 0.159 | 100% |
| **Observations (thinned)** | 12 | 136 | 49 | 0 | **197** |
| %total observations | 6% | 69% | 25% | 0% | 100% |
